# Circulating Peptidome to Indicate the Tumor-resident Proteolysis

**DOI:** 10.1038/srep09327

**Published:** 2015-03-19

**Authors:** Zaian Deng, Yaojun Li, Jia Fan, Guohui Wang, Yan Li, Yaou Zhang, Guoping Cai, Haifa Shen, Mauro Ferrari, Tony Y. Hu

**Affiliations:** 1 School of Life Science, Tsinghua University, Beijing 100084, China; 2 Department of Nanomedicine, Houston Methodist Research Institute, 6670 Bertner Avenue R8-213, Houston, TX 77030, United States; 3 Life Science Division, Graduate School at Shenzhen, Tsinghua University, Shenzhen 518055, China; 4 Institute of Biophysics, Chinese Academy Of Sciences, 15 Datum Road, Chaoyang District, Beijing 100101, China; 5 Department of Cell and Developmental Biology, Weill Cornell Medical College of Cornell University, 445 E. 69th Street, New York, New York 10021, United States; 6 Department of Internal Medicine, Weill Cornell Medical College of Cornell University, 445 E. 69th Street, New York, New York 10021, United States

## Abstract

Tumor-resident proteases (TRPs) are regarded as informative biomarkers for staging cancer progression and evaluating therapeutic efficacy. Currently in the clinic, measurement of TRP is dependent on invasive biopsies, limiting their usefulness as monitoring tools. Here we identified circulating peptides naturally produced by TRPs, and evaluated their potential to monitor the efficacy of anti-tumor treatments. We established a mouse model for ovarian cancer development and treatment by orthotopic implantation of the human drug-resistant ovarian cancer cell line HeyA8-MDR, followed by porous silicon particle- or multistage vector (MSV) - enabled EphA2 siRNA therapy. Immunohistochemistry staining of tumor tissue revealed decreased expression of matrix metallopeptidase 9 (MMP-9) in mice exhibiting positive responses to MSV-EphA2 siRNA treatment. We demonstrated, via an *ex vivo* proteolysis assay, that C3f peptides can act as substrates of MMP-9, which cleaves C3f at L_1311_-L_1312_ into two peptides (SSATTFRL and LWENGNLLR). Importantly, we showed that these two C3f-derived fragments detected in serum were primarily generated by tumor-resident, but not blood-circulating, MMP-9. Our results suggested that the presence of the circulating fragments specially derived from the localized cleavage in tumor microenvironment can be used to evaluate therapeutic efficacy of anti-cancer treatment, assessed through a relatively noninvasive and user-friendly proteomics approach.

Proteases play prominent roles in tumor growth, angiogenesis, invasion, and metastasis. Their activities can be used to monitor and evaluate responses to primary and adjuvant therapy in patients^1^,^2^. In addition, aberrant expression of some proteases within the tumor microenvironment has been examined as cancer diagnostic and prognostic biomarkers^1^,^3^,^4^. Traditionally, biopsy and blood testing are two assessment methods used to correlate the concentration of a particular protease with specific cancer morphologies. Biopsies, although a more direct assessment of tumor-associated changes, is limited in clinical application due to its invasiveness, the requirement for trained staff, and the potential to overlook metastasis^5^. Blood sampling is minimally invasive and has a substantially reduced processing time, but a relatively high detection limit and some measure of non-specificity can stymie the accuracy of standard tests such as enzyme-linked immunosorbent assay (ELISA). Since tumor-associated proteases often leach into the bloodstream at very low concentrations, ELISA testing to detect blood biomarker fluctuations is not an ideal strategy.

To overcome ELISA detection limitations for proteases, researchers have started developing methods to measure the proteases levels and activities via their proteolysis characteristics. Some have developed new output signal “amplification” approaches whereby a specific endogenous protease activity is detected by measuring the digested products of spiked, synthetic substrates in collected blood specimens^6^,^7^. These proteolytic fragments can be detected by mass spectrometry (MS), and their peak intensities reflect the blood concentration of their respective proteases – an area of study coined “exogenous peptidomics”^7^. Reliable data quality depends on many factors including the substrate concentration, incubation time, and the composition of reaction buffers. Recently, Kwong *et al*^8^ invented a nanoworm-based assay to study the proteolytic activity of matrix metalloproteinases at the tumor site. Synthetic, mass-encoded peptides conjugated to the surface of nanoworms were administered to mice, and specific cleavage of the peptides by MMPs was monitored via urinary output. By analyzing the MS profiles of the substrates collected from urine, the investigators were able to identify the dysregulated activity of tumor-resident MMPs. While this platform shows tremendous promise, it is difficult to implement in the clinic since it requires exogenously administered peptides.

“Endogenous Peptidomics”, also known as the profiling of native polypeptides in biofluids, can be mined for information pertaining to changes in protease expression and activity^9^,^10^. This approach relies on the notion that endogenous peptides act as substrates for proteases and that the presence of their proteolytic fragments accurately reflects the abundance of specific *in vivo* proteases. We hypothesize that the peptidomic signatures produced by tumor-resident proteases and host protein/peptide substrates in blood can be used to indicate the variation in target protease expression during anti-cancer treatment, and further to evaluate the therapeutic efficacy of the treatments. We established a mouse model of ovarian cancer with traceable phenotypes induced via orthotopic implantation of the human ovarian cancer cell line HeyA8-MDR. This cell line is resistant to several anti-ovarian cancer drugs such as taxol, cisplatinum, or macitentan^11^. It has been well-known that the ephrin type-A receptor 2 (EphA2), a member of the ephrin family of receptor tyrosine kinases, is overexpressed in various cancer cell types and plays an important role in tumor growth, invasiveness, angiogenesis, and metastasis through the EphA2 signaling pathway. Previously, we showed that EphA2-siRNA delivered by multistage vectors (MSV-EphA2) accumulated in tumor tissues and induced apoptosis in ovarian cancer cells^12^. In this study, we demonstrated by immunohistochemistry (IHC) analysis a significant down-regulation of MMP-9 in tumors from mice treated with MSV-EphA2, compared to animals receiving only MSV carrying control siRNA (non-silencing scramble siRNA). MMP-9 is recognized as a prognostic biomarker for treatment outcome because it is expressed at significantly higher levels in advanced ovarian tumors compared to benign tumors^13^,^14^. We confirmed that the expression level of MMP-9 is correlated with the regulation of EphA2 during treatment for the ovarian cancer. Our results suggest that two circulating peptides derived from C3f, a substrate of MMP-9 in the tumor microenvironment, reflect the content of MMP-9 in tumors, and consequently indicate the therapeutic efficacy (i.e., treatment with EphA2-siRNA). A schematic representation of this approach is depicted in Figure 1.

## Results

### Treatment with MSV-EphA2 siRNA suppresses MMP-9 expression in tumors

**Figure 2:**
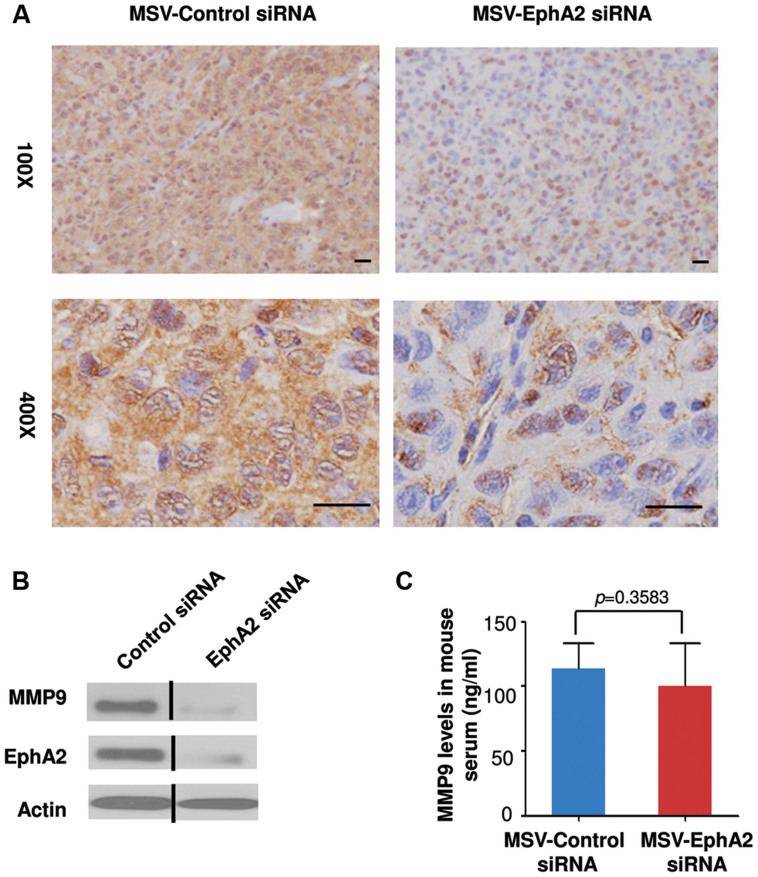
Administration or treatment with MSV-EphA2 siRNA decreased MMP-9 expression in tumors of mice with ovarian cancer and in cell cultures. (A) Immunohistochemistry analysis of tumor tissue samples from mice treated with MSV-EphA2 siRNA probing for MMP-9 expression, Upper panels 10× magnification, lower panels 40× magnification. Scale bar = 20 μm. (B) Immunoblotting to examine the effect of EphA2 siRNA transfection in HeyA8-MDR cells. Equivalent volumes of conditioned media were loaded, and the blot was probed with antibody against MMP-0, EphA2 or actin. All the blots are performed under same experimental conditions, the full image of blot as shown in Figure S1. (C) Serum MMP-9 expression in sera of tumor-bearing mice showed no statistically significant difference between control and EphA2 siRNA treatments.

EphA2 is a receptor tyrosine kinase often overexpressed in many human cancers. Previous studies have shown the correlation of EphA2 overexpression with high expression of epithelial MMP-9 enzymes in clinical samples of ovarian cancer^15^. By using siRNA knock-down of EphA2, via delivery within MSV (referred to as MSV-EphA2 siRNA in this work), Shen *et al* have shown a reduction in EphA2 expression and attenuated tumor growth in the HeyA8-MDR-induced mouse model of ovarian cancer^12^. To explore whether MSV-EphA2 siRNA treatment can likewise decrease the expression of MMP-9 in HeyA8-MDR-induced ovarian tumors, and whether the reduction is also correlated with decreased tumor burden, we performed IHC using a specific anti-human MMP-9 antibody on sections of tumor tissues collected from mice treated with either MSV-EphA2 or MSV-control siRNA. We observed a decrease in MMP-9 expression only in tumors treated with MSV-EphA2 siRNA (Figure 2A). Immunoblotting experiments confirmed that introducing (via transfection) EphA2 siRNA into HeyA8-MDR cell lines reduced the amount of MMP-9 found in the culture supernatant (Figure 2B). These results suggest that MMP-9 lies downstream of EphA2 in a signaling cascade affecting tumor growth. We did not detect MMP-9 (using anti-human MMP-9 antibody) or register any significant change (using anti-mouse MMP-9) in the serum of tumor-bearing mice (Figure 2C).

### Characterizing the proteolysis of C3f by recombinant and *in vivo* MMP-9

To identify peptides in circulation, serum peptides from HeyA8-MDR tumor-bearing mice were subjected to on-chip fractionation, followed by liquid chromatography-tandem mass spectrometry (LC-MS/MS) analysis. Detected fragments of C3f are listed in Table S1. Previous studies have speculated that C3f undergoes enzymatic hydrolysis by endoproteases and exoproteases (e.g., carboxypeptidase N)^9^,^10^; however, whether MMP-9 plays a role in this catalysis had not been determined yet. To investigate whether C3f is a substrate of MMP-9, we designed and synthesized C3f harboring two 6xHis tags, one located at each terminus of the protein fragment (6xHis-C3f_1304-1320_-6xHis), to protect C3f from degradation. As described in Materials and Methods, this synthetic C3f was incubated with recombinant MMP-9 prior to matrix-assisted laser desorption/ionization time-of-flight mass spectrometry (MALDI-TOF MS) analysis. New peaks appeared in the MS spectrum only when MMP-9 and the 6xHis-C3f_1304-1320_-6xHis peptides were combined. Peak sequences were matched to the sequence of synthetic C3f using the FindPept tool in ExPASy (http://web.expasy.org/findpept/). Several cleavage sites (R_1310_–L_1311_, L_1311_–L_1312_, W_1313_–E_1314_, and N_1317_–L_1318_) were identified in synthetic C3f, and these were also found in endogenous C3f (Figure S2). Two of these specific proteolytic products, which we now strongly presume to be MMP-9-catalyzed, appeared as MS peaks with mass/charge (m/z) ratio of 1704.8 Da and 1936.9 Da (Figure S3A). Using MALDI-TOF/TOF MS analysis, we confirmed their amino acid sequences to be 6xHis-SSATTFRL (m/z = 1704.8) and LWENGNLLR-6xHis (m/z = 1936.9). Indeed, their daughter fragments also presented in the individual MS-MS spectrum (Figure S3B). These results confirm that C3f is an enzymatic substrate of MMP-9.

**Figure 3:**
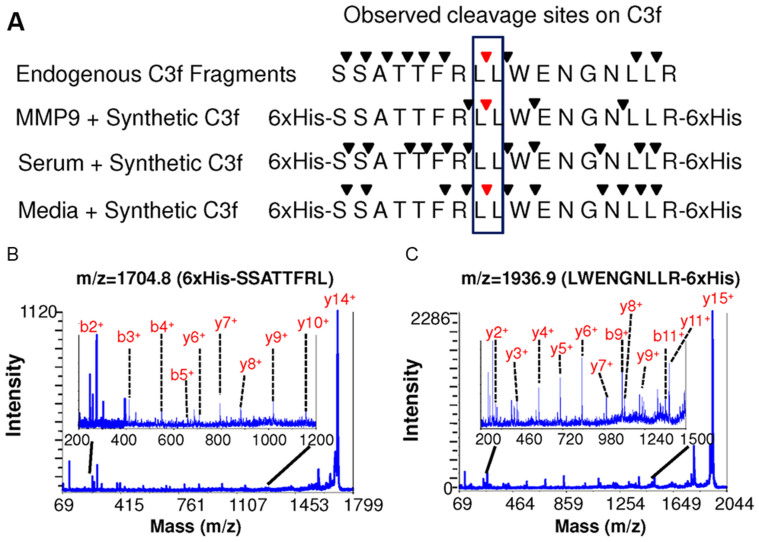
Comparison of natural C3f fragments and the cleavage site analysis after incubation with MMP-9, serum, and cell culture media indicate tumor protease specificity. (A) Observed cleavage sites on C3f. Arrows show the observed cleavage sites. Red arrows show the cleavage sites of interest. The series of b- and y-ions in MALDI-TOF/TOF MS spectrum confirmed the amino acid sequence of these two peaks as (B) 6xHis-SSATTFRL (m/z = 1704.8) and (C) LWENGNLLR-6xHis (m/z = 1936.9). Insets show the zoomed-in view of the MS-MS spectra.

Because C3f, as a fragment of complement C3, exists throughout the blood circulation and within tumor tissues, it remains unclear whether other protease(s) among possibly hundreds in biofluids, in addition to MMP-9, could also specifically cleave C3f^4^. To investigate this prospect in such a complex milieu, we incubated synthetic C3f peptides with serum from tumor-bearing mice, treated with MSV-control siRNA or conditioned media of HeyA8-MDR, prior to MALDI-TOF MS analysis. Each newly generated peak with specific m/z was assigned to a unique sequence (both listed in Table S1). Both 6xHis-SSATTFRL and LWENGNLLR-6xHis were only found as proteolytic products when synthetic C3f was incubated with conditioned media, and not with mouse serum (Figure S3). Collectively, as shown in Figure 3, these results indicate that the cleavage on peptide substrate is dependent on its environment (i.e., in complex serum or more simplified cell culture medium), where the presence of other proteases and/or factors may affect proteolytic activity. And these results suggest that the endogenous cleavage products of C3f at L_1311_–L_1312_ in serum are processed by tumor cell secreting MMP-9.

### Enzymatic cleavage of C3f in conditioned media depends on MMP-9 expression

**Figure 4:**
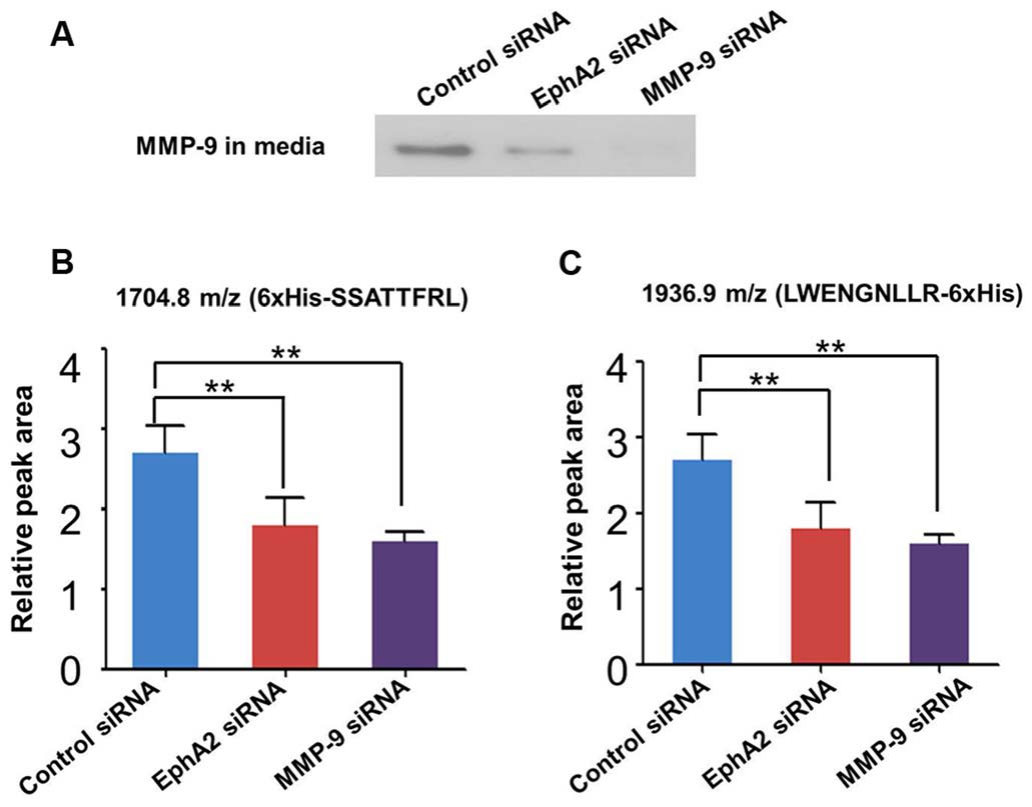
MMP-9 expression in cell culture media correlates with the extent of cleavage of the L_1311_ – L_1312_ peptide bond in C3f. (A) Immunoblotting using equivalent sample volumes of conditioned media from cells transfected with control siRNA, EphA2 siRNA, or MMP-9 siRNA. To confirm MMP-9 expression levels. All gel and blots run under same condition, the full image as shown in Figure S4. (B) The relative peak intensities of two specific fragments, 6xHis-SSATTFRL (m/z = 1704.8) and (C) LWENGNLLR-6xHis (m/z = 1936.9), generated by incubating synthetic C3f with culture media under the same conditions indicated in (A). **, p < 0.01.

Based on our results described in the previous section, C3f is cleaved by MMP-9 at L_1311_ –L_1312_ in cell-conditioned media, but whether this protease is predominantly (or solely) responsible for C3f proteolysis under this circumstance remains to be explored. To this end, we measured the yield of C3f-derived fragments (i.e., 6xHis-SSATTFRL and LWENGNLLR-6xHis) in HeyA8-MDR-conditioned media, using MALDI-TOF MS subsequent to peptide on-chip fractionation. Cleavage kinetics assays revealed that yields of 6xHis-SSATTFRL and LWENGNLLR-6xHis reached saturation by 4 hours (Figure S5). Moreover, we demonstrated that the generation of 6xHis-SSATTFRL and LWENGNLLR-6xHis decreased as a function of MMP-9 inhibition via introduction of EphA2 siRNA or MMP-9 siRNA (Figure 4). These observations suggest that MMP-9 is the major protease responsible for cleavage of C3f at the L_1311_ –L_1312_ position and that this activity also depends on the expression of EphA2.

### Relative quantification of C3f cleavage products in serum

**Figure 5:**
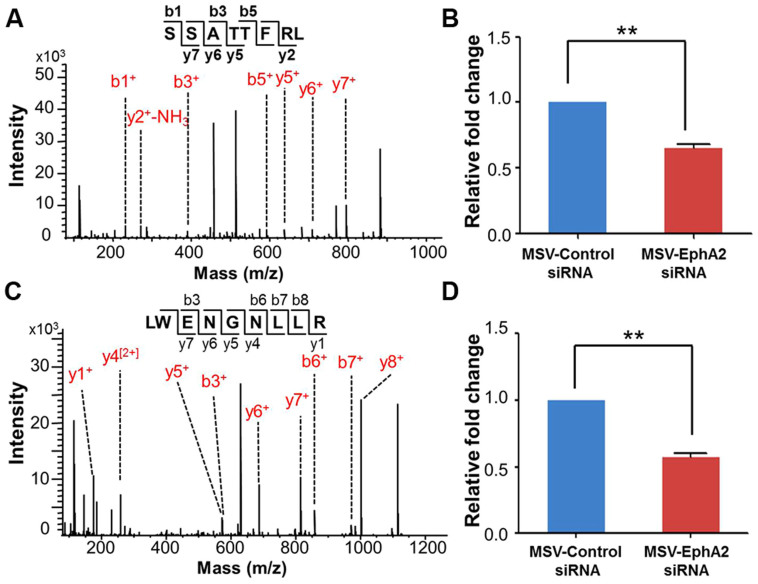
Peptide sequences and the relative expression levels of the two C3f fragments in serum (SSATTFRL and LWENGNLLR) were determined and quantified using iTRAQ. Serum peptides from mice treated with MSV-control siRNA were labeled with 114, whereas serum peptides from mice treated with MSV-EphA2 siRNA were labeled with 115. (A) The b- and y-ion series indicate the sequence of SSATTFRL, and a zoomed-in view (B) shows its reporter ion region at m/z = 114.1 and 115.1. (C) The b- and y-ion series indicate the sequence of LWENGNLLR, and a zoomed-in view (D) shows its reporter ion region at m/z = 114 and 115.

For *in vivo* validation and quantification of fragment-specific C3f cleavage, we measured the relative level of SSATTFRL and LWENGNLLR in serum from mice treated with MSV-control siRNA or MSV-EphA2 siRNA, using the isobaric tags for relative and absolute quantification (iTRAQ) approach. Equivalent volumes of sera from the two treatment groups were processed through on-chip fractionation. Both the intraday and interday coefficients of variance (CVs) were less than 30% (Table S3, S4), indicating good reproducibility for the fractionation process. Enriched and eluted peptides were labeled as follows: 114 reporter tag for peptides from mice treated with MSV-control siRNA; 115 reporter tag for peptides from mice treated with MSV-EphA2 siRNA. The series of b- and y-ions in the MS-MS spectra, fragments of the precursor ions, confirmed the fragments SSATTFRL and LWENGNLLR to be detectable in mice of both treatment groups (Figure 5A and 5C). However, a comparison of the intensities of the reporter tags revealed less detectable SSATTFRL and LWENGNLLR fragments in mice treated by MSV-EphA2 siRNA, with a ratio of 0.64 and 0.55, respectively (Figure 5B and 5D). Moreover, the levels of the parent protein, complement C3, exhibited no significant difference between the MSV-EphA2 and MSV-control siRNA treatment groups (Figure S6) measured by ELISA. These results indicate that different levels of these two peptides generated by MMP-9 correlate with EphA2 siRNA treatment.

## Discussion

Tumor development may be characterized by molecular changes (e.g., enzymatic activities, protein shedding, extracellular matrix remodeling, etc.) in tumor cells or the tumor microenvironment prior to noticeable physiological symptoms^16^,^17^. Small peptides secreted by cells, shed from cell surfaces, or cleaved by tumor-resident proteases, are more likely to enter the bloodstream where they can be quickly and easily surveyed as part of an early diagnostic or prognostic strategy for cancer care^18^. As such, the circulating peptidome holds tremendous diagnostic and therapeutic value, particularly in the face of challenges such as tumor heterogeneity and the development of drug resistance in cancer therapy. Moreover, proteins/peptides in biofluids often tell a story about their catalytic partnering protease(s)^19^,^20^.

To verify our hypothesis that circulating peptides (i.e., C3f-derived fragments) can function as reliable indicators of a cancer-associated proteolytic event for prognostic purposes, we employed an ovarian cancer mouse model that can be induced through implantation of the docetaxel-resistant ovarian cancer cell line HeyA8-MDR. Historically, EphA2 was considered a potential therapeutic target for the treatment of ovarian cancer via RNA interference^21^. However, until recently, the lack of an effective siRNA delivery mechanism dramatically limited anti-EphA2 in *in vivo* applications. Previously, we developed an MSV system composed of discoidal porous silicon particles loaded with nanotherapeutics, for targeted delivery and sustained release of siRNA to tumors. Using chemotherapy-sensitive/resistant mouse models, we demonstrated that treatment with MSV-EphA2 siRNA significantly decreased the tumor burden usually characteristic of HeyA8-MDR-induced tumor-bearing mice^12^. Others have reported that inhibition of EphA2 expression may down-regulate expression of MMP-9 in a murine choroidal melanoma model, and PI3K may be the mediator in this regulatory pathway^22^. In this study, our IHC and immunoblotting results show that the decreased expression of MMP-9 in HeyA8-MDR-induced tumors could serve as an indicator of ovarian cancer treatment efficacy.

Traditional methods such as IHC, ELISA, immunoblotting and zymography have been used to determine the variation of MMP-9 expression and activity as a result of cancer development^1^. Such methods may readily detect the amount of enzyme within the tumor microenvironment; however, they prove inadequate at measuring low-level enzymes secreted into circulation. Thus, currently, invasive techniques such as tumor biopsies are still needed for complete diagnosis and treatment monitoring. The reliability of the biopsy method is limited by sample localization in tissue and physician experience, both contributing to the inadequacy of the method for treatment monitoring. In this study, we suggest a different approach by examining the expression of MMP-9 within the tumor microenvironment, as well as in the blood circulation of mice bearing HeyA8-MDR-induced tumors. The peptide fragments generated by tumor-resident MMP-9 and released into the bloodstream – their quantities and identities presented in our comprehensive analyses here – strongly correlate with enzymatic expression and reflect the treatment effect using a novel nanovector-enabled siRNA delivery strategy.

Another challenge is isolating these low-molecular-weight peptides from serum. Serum proteins and peptides can exist within a large dynamic range of concentrations, and the blood circulation is dominated by larger and highly abundant proteins such as immunoglobulin, albumin, and transferrin^23^. Previously we demonstrated our ability to optimize the morphologies of nanoporous silica films by engineering the physicochemical properties (pore size, pore structure, and surface affinity, etc.), all of which can selectively enrich low-molecular-weight and low-abundant peptides from serum samples^24^,^25^,^26^. Applying this peptide on-chip fractionation coupled to MALDI-TOF MS analysis, we can directly profile and investigate the activity of MMP-9 and its cleavage products (peptide fragments) in blood and in cancer cell cultures. Here, we show that C3f is specifically cleaved into two peptides (SSATTFRL and LWENGNLLR) by MMP-9 at the L_1311_–L_1312_ position, in cancer cell-conditioned culture media. This scenario mimics the tumor microenvironment *in vivo* and is often used as a model in proteomics studies^27^. This cleavage event can be found only in conditioned media and not in the serum of tumor-bearing mice, suggesting that the cleavage occurred as a result of action by MMP-9 resident in the tumor microenvironment, rather than enzymes circulating in blood.

Loss-of-function studies using small interfering RNA (siRNA) comprise a powerful method to evaluate gene function in a particular biology process^28^. In this study, we showed that the culture media of HeyA8-MDR cells transfected with EphA2 siRNA or MMP-9 siRNA have lower cleavage capacity at the L_1311_-L_1312_ site of synthetic C3f than the media of the cells transfected with only control siRNA, which strongly suggests MMP-9 is the major protease secreted from HeyA8-MDR cells that hydrolyzes C3f. To further validate that the two identified C3f fragments can be the proteolytic products of endoproteases in the tumor microenvironment, iTRAQ labelling and LC-MS/MS were applied to relatively quantify the concentrations of these two fragments in mouse serum. Our data shows that both of the fragments were decreased 1.6 folds in sera of mice treated with MSV-EphA2 siRNA, compared to animals treated with MSV-control siRNA. Cumulatively, our results demonstrate that these two C3f-derived peptides are reflective of MMP-9 expression in tumor cells and can potentially be used as measures of EphA2 treatment efficacy.

Human and murine proteases can be classified into four groups usually according to their substrates – aspartic proteases, cysteine proteases, serine proteases and metalloproteases^4^. Proteases from each category cleave their substrates at specific sites. However, there are a number of proteolytic events occurring at any one time in the body, and they largely depend on protease activation, inhibition, and processing^29^. Circulating peptidomics constitutes one of the hotly-debated research fields, in part, due to its enormous potential to expand the scope of biomarkers research and application^9^,^10^. Peptides in serum are not considered as *bona fide* biomarkers but as reflections of the expression and/or activities of their corresponding proteases within the tumor microenvironment. In this paper, we present strong evidence that the generation of circulating peptide fragments is dependent on expression/activity of tumor-resident endoproteases. These peptides can be detected and measured using a powerful and virtually non-invasive proteomics approach. We view this as a promising alternative strategy to discover novel circulating biomarkers, and provide a map for tracing cancer-specific metabolic or immunological signatures indicative of tumor progression and treatment response with a “higher resolution”.

## Methods

### Cell culture and conditioned media collection

HeyA8-MDR cells were maintained in RPMI-1640 media supplemented with 15% fetal bovine serum (FBS) in 5% CO_2_/95% air at 37°C. Once the HeyA8-MDR cells reached 70–80% confluency in 10 cm petri dishes, the spent media were discarded and cells rinsed three times with 1× phosphate-buffered saline (PBS). Then, 5 mL of serum-free medium were added, and the cells were incubated for an additional 24 hr. The conditioned media (CM) were subsequently collected by centrifugation to remove cell debris. One ml of HeyA8-MDR-conditioned medium was concentrated with 3 KD Amicon Ultra-0.5 mL centrifugal filters (Millipore) to 50 μl.

### C3f cleavage assay with MMP-9

Synthetic C3f peptides (50 μM) were incubated with MMP-9 (Millipore) in 50 mM HEPES with 10 mM CaCl_2_. Synthetic C3f or recombinant MMP-9 alone in reaction buffer served as the controls. After on-chip fractionation (method described below in “On-chip fractionation”), eluted peptide fragments were analyzed using matrix-assisted laser desorption/ionization time-of-flight mass spectrometry (MALDI-TOF MS). The individual peaks, representing fragments presumably generated by proteases resident in conditioned cell culture media and/or mouse sera, were matched to synthetic, known C3f sequences by FindPept tools in ExPASy (http://web.expasy.org/findpept/).

### C3f cleavage assay with serum or conditioned media

Sera from tumor-bearing mice, collected from animals treated with MSV-EphA2 siRNA or MSV-control siRNA, were diluted 10 times with 1× PBS buffer. Then, 1 μl of each serum sample (to minimize interference from abundant serum peptides) or 10 μl of concentrated cell culture medium was added separately to the reaction buffer (50 mM HEPES with 10 mM CaCl_2_). Next, synthetic C3f peptides were added to each reaction buffer sample containing serum or conditioned medium to reach 50 μM (final concentration), and mixtures were incubated at 37°C for 3 hrs. Mouse sera from each treatment group, conditioned-medium samples, or synthetic C3f peptides were added to the reaction buffers individually to serve as control reactions. Peptide cleavage products from each reaction were purified and detected as described above in “C3f cleavage assay with MMP-9”. To conduct the relative quantification of the peptides of interest, an adrenocorticotropic hormone (ACTH) fragment (ACTH_18–39_, RPVKVYPNGAEDESAEAFPLEF, monoisotopic mass at 2464.2 Da) at 50 nM, serving as an internal standard, was mixed in elution buffer prior to extraction from nanopores and analysis by MS. The relative peak areas were calculated by dividing the peak area of target peptides by the peak area of ACTH_18–39_.

### On-chip fractionation

On-chip fractionation via nanoporous silica chips was conducted to isolate and enrich serological peptides from ovarian cancer mice sera or HeyA8-MDR-conditioned media. The fractionation protocol was developed and has been extensively described by our group^10^. To each serum sample (20 μl) thawed on ice, 1.7 μl of a 5% Trifluoroacetic acid (TFA) solution was added, mixed by vortexing, and pH-adjusted to approximately 5. Next, each serum-TFA mixture (5 μl) was pipetted on top of a multi-welled mesoporous silica chip, in duplicate wells. The chip was covered to prevent evaporation and incubated at 25°C in a humidified chamber for 30 min. Chip processing proceeded by washing with deionized water to remove large proteins, and eluting the bound peptides with 6 μl of elution buffer (50% acetonitrile and 0.1% TFA). To assess the intraday and interday variability, three mouse serum samples underwent on-chip fractionation, and the eluates were analyzed using MALDI-TOF MS. Five representative peaks with different m/z and different intensities were selected as characteristic peaks to calculate the coefficient of variation (CV).

### Relative quantification of peptide markers using iTRAQ labeling

Isobaric tags for relative and absolute quantification (iTRAQ) was used to confirm the relative level of peptide expression in serum of ovarian tumor-bearing mice. All of the mouse serum samples (15 μl each) from each treatment group (injected with MSV-control siRNA or MSV-EphA2 siRNA) were pooled. To each serum pool, 10.2 μl of 5% TFA was added, and the peptides in solution were isolated by on-chip fractionation. The eluates from the group with the same treatment were combined, dried under vacuum centrifugation, and reconstituted in 0.5 M tetraethylammonium bromide (TEAB). Each of the iTRAQ reagents (AB-Sciex, Foster City, CA) was dissolved in 70 μl of ethanol for 2 min with vortexing and added directly to the dissolved peptides. The samples were mixed by vortexing and incubated for one hour at 25°C. The derivatized peptides were then pooled, dried under vacuum centrifugation, and re-dissolved in 0.1% TFA/water. Following desalting on a small C_18_ column (2 mm × 1 cm), the peak fractions were left to air dry before being re-dissolved in buffer (1% formic acid in water containing 5 mM ammonium acetate). The pooled, derivatized and desalted peptides were subjected to LC-MS/MS analysis on a LTQ-Orbitrap Elite. The data were analyzed and quantified using Mascot (Matrix Science, London, UK, ver. 2.3) via Proteome Discoverer (Thermo Scientific, version 1.3).

### Statistical analysis

The two-tailed student's t test was used to calculate the significance of differences observed across experimental samples. A *p* value less than 0.05 was considered statistically significant.

## Author Contributions

T.H. is the principal investigator who proposed and organized the study. T.H., M.F., Y.Z., G.C., Y.L. and Z.D. designed this study; H.S. provided the mouse model; Z.D. and J.F. collected serum samples; G.W. participated in the western blotting; Z.D. analyzed data, Z.D., Y.L. and J.F. drafted the main manuscript. All authors read the final manuscript.

## Supplementary Material

Supplementary Information Supplementary Information

## Figures and Tables

**Figure 1:**
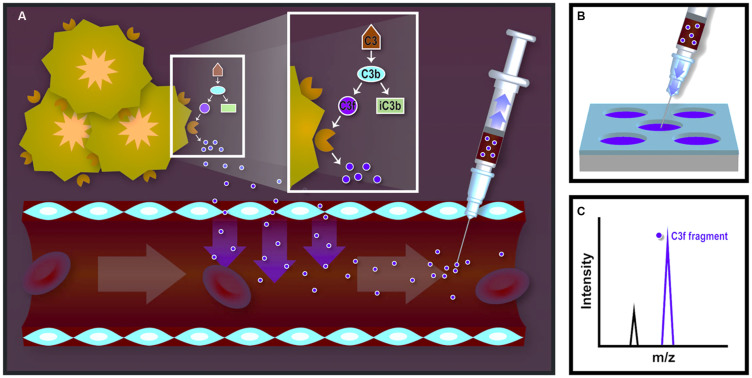
A schematic representation of peptidomics analysis for blood biomarkers of tumor activity. (A) Proteins/peptides are cleaved by proteases within the vicinity of tumor tissues, correlating with tumor growth or response to treatment. These disease-associated proteolytic fragments are released into the bloodstream. (B) Blood/serum samples are processed through on-chip fractionation with nanopore platforms to enrich low-molecular weight peptides. (C) The target biomarkers (proteolytic products of tumor-resident proteases) are detected with mass spectrometry. The specific peptide peaks in the mass spectrum represent the expression and/or activity of their corresponding tumor-resident peptidases.
