# Supplementary material for: Circulating Peptidome to Indicate the Tumor-resident Proteolysis
Source: Sci Rep. 2015 Mar 19;5:9327. doi: 10.1038/srep09327 (PMC4365414; doi:10.1038/srep09327)
Supplement: Supplementary Information [file srep09327-s1.doc]

**Circulating Peptidome to Indicate the Tumor-resident Proteolysis**

Zaian Deng^1,2,3^, Yaojun Li^2^, Jia Fan^2^, Guohui Wang^2^, Yan Li^4^, Yaou Zhang^1,3^, Guoping Cai^1,3^, Haifa Shen^2,5^, Mauro Ferrari^2,6^, Ye Hu^2,5🖂^

^1^School of Life Science, Tsinghua University, Beijing 100084, China,

^2^Department of Nanomedicine, Houston Methodist Research Institute, 6670 Bertner Avenue R8-213, Houston, TX 77030, United States,

^3^Life Science Division, Graduate School at Shenzhen, Tsinghua University, Shenzhen 518055, China,

^4^Institute of Biophysics, Chinese Academy Of Sciences, 15 Datum Road, Chaoyang District, Beijing 100101, China

^5^Department of Cell and Developmental Biology, Weill Cornell Medical College of Cornell University, 445 E. 69th Street, New York, New York 10021, United States,

^6^Department of Internal Medicine, Weill Cornell Medical College of Cornell University, 445 E. 69th Street, New York, New York 10021, United States

# Supplementary Materials and Methods

## Serum collection

The mouse model of ovarian cancer used in these studies was established as described previously . Briefly, HeyA8-MDR cells were implanted into the peritoneal cavity of nude mice to initiate tumor growth (n = 30). Two weeks after implantation, 20 mice were randomly selected and assigned to different treatment groups, each group treated biweekly with either MSV-control siRNA (non-silencing scramble siRNA) (n = 10) or MSV-EphA2 siRNA (n = 10). The mice sera were collected by retro-orbital bleeding after 6 weeks of treatment. The blood samples were kept at 25°C for 1 hr and then centrifuged at 4000 x g for 15 min. Sera were carefully collected and then stored at -80°C in aliquots until use. The animal studies were performed in accordance with the guidelines of the Animal Welfare Act and the Guide for the Care and Use of Laboratory Animals following protocols which were approved by the Institutional Animal Care and Use Committee (IACUC).

## Immunohistochemistry analysis

Immunohistochemistry (IHC) analysis was conducted as previously described . Paraffin-embedded tumor tissue samples were stripped of the paraffin and subsequently probed with rabbit polyclonal anti-human MMP-9 antibodies (dilution 1:100, Millipore). After multiple washes, sample slides were incubated with horseradish peroxidase (HRP)-conjugated secondary antibody followed by diaminobenzidine (DAB) treatment for signal detection.

## siRNA transfection

The sequences of non-silencing scramble siRNA (control), EphA2 siRNA, and MMP-9 siRNA (purchased from Santa Cruz Biotechnology) were previously described . The protocol for siRNA transfection was conducted as follows: An equivalent number of HeyA8-MDR cells was plated in culture and incubated in 5% CO_2_ until the cells reached 60-80% confluency in each well of a 6-well dish. Cells were then washed with siRNA transfection media (Santa Cruz Biotech) and treated with control EphA2, or MMP-9 siRNA following the manufacturer’s siRNA transfection protocol (Santa Cruz). Following a 48-hour incubation, the transfected cells were washed three times with PBS, supplemented with 1 ml of RPMI-1640 serum-free medium, and incubated for an additional 18 hr. Cellular proteins were extracted with RIPA buffer (Thermo Scientific) following the manufacturer’s protocol. One ml of conditioned medium was concentrated with 3-KD Amicon Ultra-0.5 mL centrifugal filters to 50 μl. An equivalent volume of each conditioned-medium sample from the various siRNA transfections was subjected to western immunoblot analysis and enzyme cleavage. Cleavage analysis of synthetic C3f in conditioned media was also performed, following the cleavage protocol described in the section “C3f cleavage assay with serum or conditioned media”.

## Enzyme-linked immunosorbent assays (ELISA) to detect MMP9 in serum

The levels of human and mouse MMP9 in serum were determined using commercial enzyme-linked immunosorbent assays kits purchased from Invitrogen Corporation and R&D Systems, Inc, respectively. ELISA kits for detecting complement component 3 were purchased from USCN Life Science Inc. ELISA assays were conducted according to the manufacturer’s instructions. Using protein standards, calibration curves were prepared and used to calculate the concentration of target proteins in serum samples collected above.

## Western immunoblotting

Cell lysate or cell-secreted proteins collected from conditioned media were separated on 12% SDS-PAGE gels and transferred (300 mA for 1 hr) to PVDF membranes. Blots were blocked at 25°C in PBST (PBS with 0.1% Tween-20) with 5% (wt/vol) nonfat milk for 2 hr. The membranes were incubated overnight with rabbit anti-human MMP-9 antibodies (Cell Signaling) or rabbit anti-β-Actin (Cell Signaling; 1:1000). After washing, membranes were then incubated with goat anti-rabbit IgG-HRP antibody (Santa Cruz Biotech) for 1 hr at 25**°**C. The blots were visualized using the ECL-Plus Detection kit (Thermo Scientific Co.).

## Kinetic assay for C3f cleavage using conditioned media

Concentrated cell culture media of HeyA8 cells were collected as described above in section “siRNA transfection”. The media were then combined with reaction buffer (50 mM HEPES with 10mM CaCl_2_) containing 50 μM synthetic C3f. This mixture was split into 7 aliquots of 140 µL each and then incubated at 37°C for 0, 1, 2, 3, 4, 5, or 6 hr to allow time for enzymatic cleavage. The reactions were heat-terminated at 50°C for 5 min. Peptides from each reaction were purified and detected using the method described above in “C3f Cleavage assay with MMP-9”.

## MALDI-TOF MS

Following on-chip fractionation, isolated peptides were spotted (0.5 μl of each eluate) in the sample well on top of disposable MALDI target plates and allowed to dry. Matrix solution (0.5 µl) consisting of 5 g/L of a-cyano-4-hydroxycinnamic acid (CHCA) in 50 % ACN and 0.1 % TFA, was spotted over the dried sample and left to crystallize at 25°C. The samples were processed on an Applied Biosystems 4700 MALDI TOF/TOF Analyzer (Applied Biosystems, Inc.), and each spectrum was recorded and introduced into the software MARKVIEW V1.2.1 to obtain peak response values.

## LC–MS/MS

To identify the amino acid sequences of chip-fractionated peptides described above, all of the eluates from the same treatment group were pooled and subjected to LC-MS/MS according to the method previously described . Prior to injection into the LC equipment, samples were dried by vacuumed centrifugation and resuspended in compatible buffer (1% formic acid, 5 mM NH4-OAc). Reversed-phase chromatography was performed on an Agilent 1200 series HPLC autosampler. Gradient solvents used for LC analysis include: (A) 0.1% formic acid in water and (B) 0.1% formic acid in acetonitrile. Peptide analysis was performed on an Orbitrap-XL mass spectrometer (Thermo Scientific, Waltham MA). Acquisition parameters include 1 FTMS scan at 60,000 resolution, followed by 3 MS/MS product ion scans (in the ion trap) of 2 microscans each (total run time equals 60 min). Mascot (v 2.3, Matrix Science, London, UK) or Sequest (Thermo, San Jose, CA) was used to compare the LC spectra of the fragments against the SwissProt (EBI) protein database for peptide sequence identification. Database search parameters were typically set to allow mass tolerances of 15 ppm, 0.8 Da for fragment ions, and up to nine missed cleavages in non-enzyme digestions. Methionine sulfoxide and pyro-glutamate formation were considered variable modifications.

**Supplementary information**

**
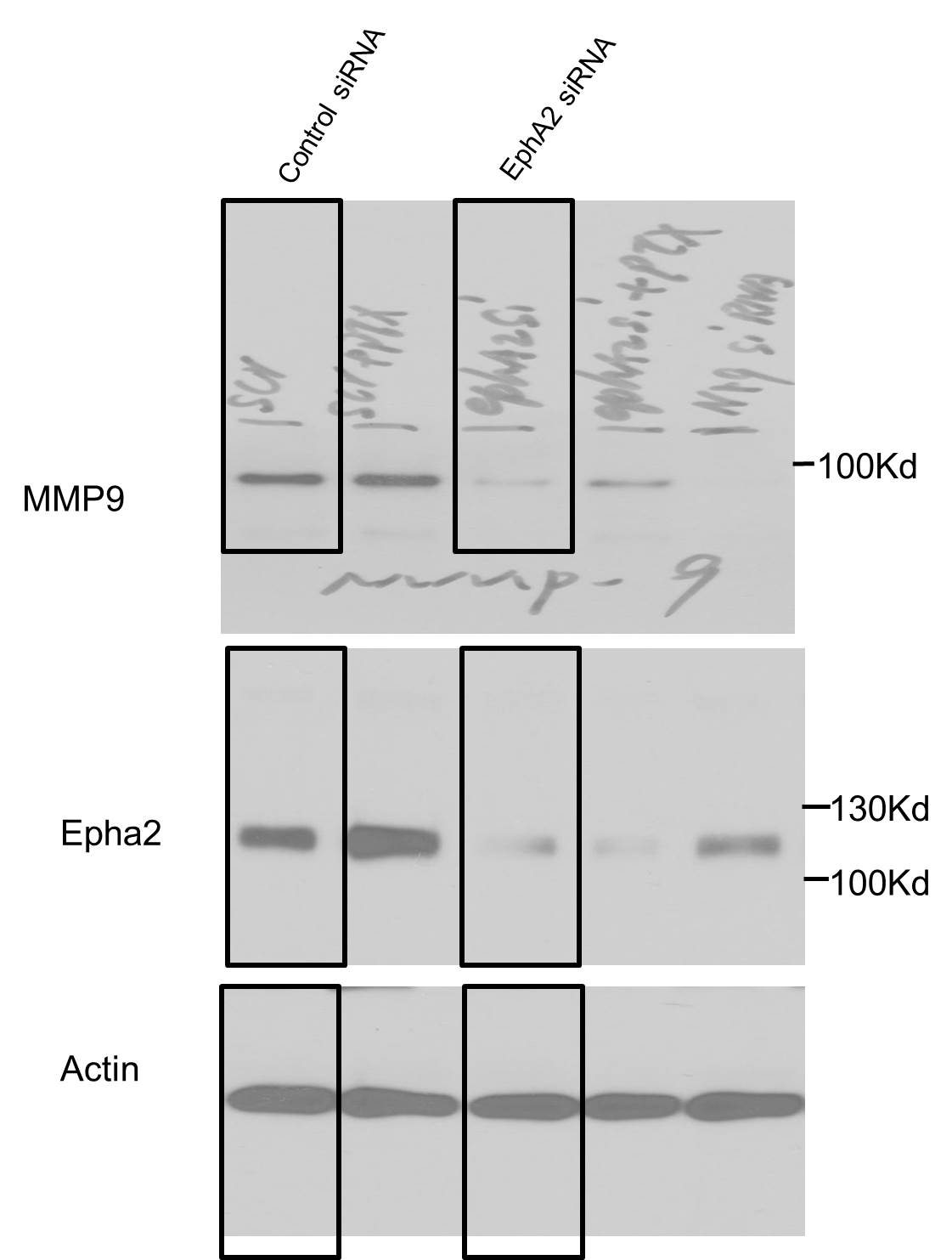
**

Figure S1 | Full western blot image show the expression level of EphA2 in cell lysate and MMP9 in cell culture media.


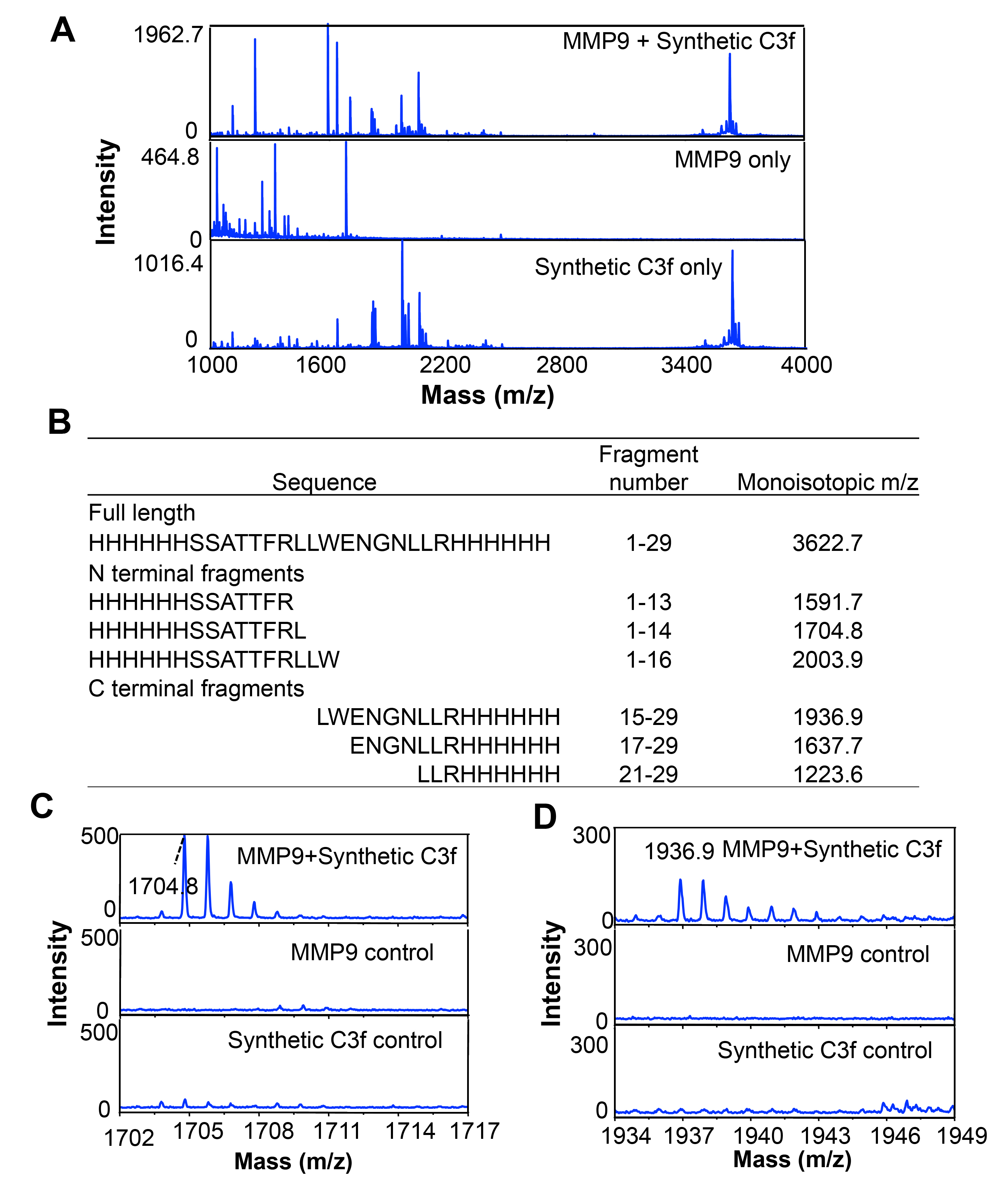


Figure S2 | Endogenous MMP-9 cleavage sites in synthetic C3f were identified using MALDI-TOF MS. (A) Wide-range view of the MALDI-TOF MS spectra showing C3f peaks generated by MMP-9. (B) Amino acid residue sequences of C3f fragments generated by incubation of synthetic C3f with MMP-9. Zoom-in view shows the L_1311_ – L_1312_ bond in synthetic C3f can be cleaved by MMP-9 into two fragments with peaks at (C) m/z=1704.8 and (D) m/z=1936.9 detected in the MALDI-TOF MS spectrum.

| Table S1. Peptides of C3f in serum could be detected using LC-MS/MS | | | |
| --- | --- | --- | --- |
| Mass of peptides | Peptide sequence | Score |  |
| 882.46 | R.SSATTFRL.L | 42 |  |
| 958.49 | L.LWENGNLL.R | 34 |  |
| 995.55 | R.SSATTFRLL.W | 47 |  |
| 1114.59 | F.RLLWENGNL.L | 42 |  |
| 1114.60 | L.LWENGNLLR.S | 44 |  |
| 1227.68 | F.RLLWENGNLL.R | 41 |  |
| 1261.66 | T.FRLLWENGNL.L | 50 |  |
| 1362.71 | T.TFRLLWENGNL.L | 41 |  |
| 1374.75 | T.FRLLWENGNLL.R | 39 |  |
| 1383.78 | F.RLLWENGNLLR.S | 36 |  |
| 1463.76 | A.TTFRLLWENGNL.L | 30 |  |
| 1475.80 | T.TFRLLWENGNLL.R | 55 |  |
| 1576.85 | A.TTFRLLWENGNLL.R | 40 |  |
| 1647.88 | S.ATTFRLLWENGNLL.R | 40 |  |
| 1708.86 | R.SSATTFRLLWENGNL.L | 40 |  |
| 1732.95 | A.TTFRLLWENGNLLR.S | 57 |  |
| 1734.91 | S.SATTFRLLWENGNLL.R | 41 |  |
| 1821.95 | R.SSATTFRLLWENGNLL.R | 41 |  |
| 1978.05 | R.SSATTFRLLWENGNLLR.S | 46 |  |
|  | | | |

| Table S2. Peaks generated in serum and cell culture medium matching synthetic C3f sequences with Findpept tool | | | | | | |
| --- | --- | --- | --- | --- | --- | --- |
| Proteolysis of C3f in serum | | |  | Proteolysis of C3f in HeyA8-MDR cell culture medium | | |
| Mass to charge | Peptide | Position |  | Mass to charge | Peptide | Position |
| 928.43 | HHHHHHS | 1-7 |  | 928.40 | HHHHHHS | 1-7 |
| 997.47 | RHHHHHH | 23-29 |  | 997.47 | RHHHHHH | 23-29 |
| 1014.45 | HHHHHHSS | 1-8 |  | 1015.44 | HHHHHHSS | 1-8 |
| 1050.46 | HHHHHSSAT | 2-10 |  | 1110.56 | LRHHHHHH | 22-29 |
| 1151.51 | HHHHHSSATT | 2-11 |  | 1223.64 | LLRHHHHHH | 21-29 |
| 1187.52 | HHHHHHSSAT | 1-10 |  | 1223.64 | SATTFRLLWE | 8-17 |
| 1227.68 | RLLWENGNLL | 13-22 |  | 1317.62 | HHHHSSATTFR | 3-13 |
| 1227.68 | LLWENGNLLR | 14-23 |  | 1337.68 | NLLRHHHHHH | 20-29 |
| 1269.67 | HHSSATTFRLL | 5-15 |  | 1337.68 | SATTFRLLWEN | 8-18 |
| 1288.59 | HHHHHHSSATT | 1-11 |  | 1435.64 | HHHHHHSSATTF | 1-12 |
| 1298.57 | HHHHHSSATTF | 2-12 |  | 1454.68 | HHHHHSSATTFR | 2-13 |
| 1310.67 | SSATTFRLLWE | 7-17 |  | 1591.74 | HHHHHHSSATTFR | 1-13 |
| 1363.67 | ENGNLLRHHHH | 17-27 |  | 1637.79 | ENGNLLRHHHHHH | 17-29 |
| 1406.73 | HHHSSATTFRLL | 4-15 |  | 1704.82 | HHHHHHSSATTFRL | 1-14 |
| 1435.64 | HHHHHHSSATTF | 1-12 |  | 1823.87 | WENGNLLRHHHHHH | 16-29 |
| 1543.79 | HHHHSSATTFRLL | 3-15 |  | 1936.95 | LWENGNLLRHHHHHH | 15-29 |
| 1591.74 | HHHHHHSSATTFR | 1-13 |  | 2050.04 | LLWENGNLLRHHHHHH | 14-29 |
| 1637.79 | ENGNLLRHHHHHH | 17-29 |  | 2206.14 | RLLWENGNLLRHHHHHH | 13-29 |
| 1657.9 | RLLWENGNLLRHH | 13-25 |  |  | | |
| 1680.85 | HHHHHSSATTFRLL | 2-15 |  |  |  |  |
| 1817.91 | HHHHHHSSATTFRLL | 1-15 |  |  |  |  |
| 2206.14 | RLLWENGNLLRHHHHHH | 13-29 |  |  |  |  |
| 2644.3 | HHHHHHSSATTFRLLWENGNLL | 1-22 |  |  |  |  |
|  | |  |  |  |  |  |
|  |  |  |  |  |  |  |
|  | | |  |  |  |  |


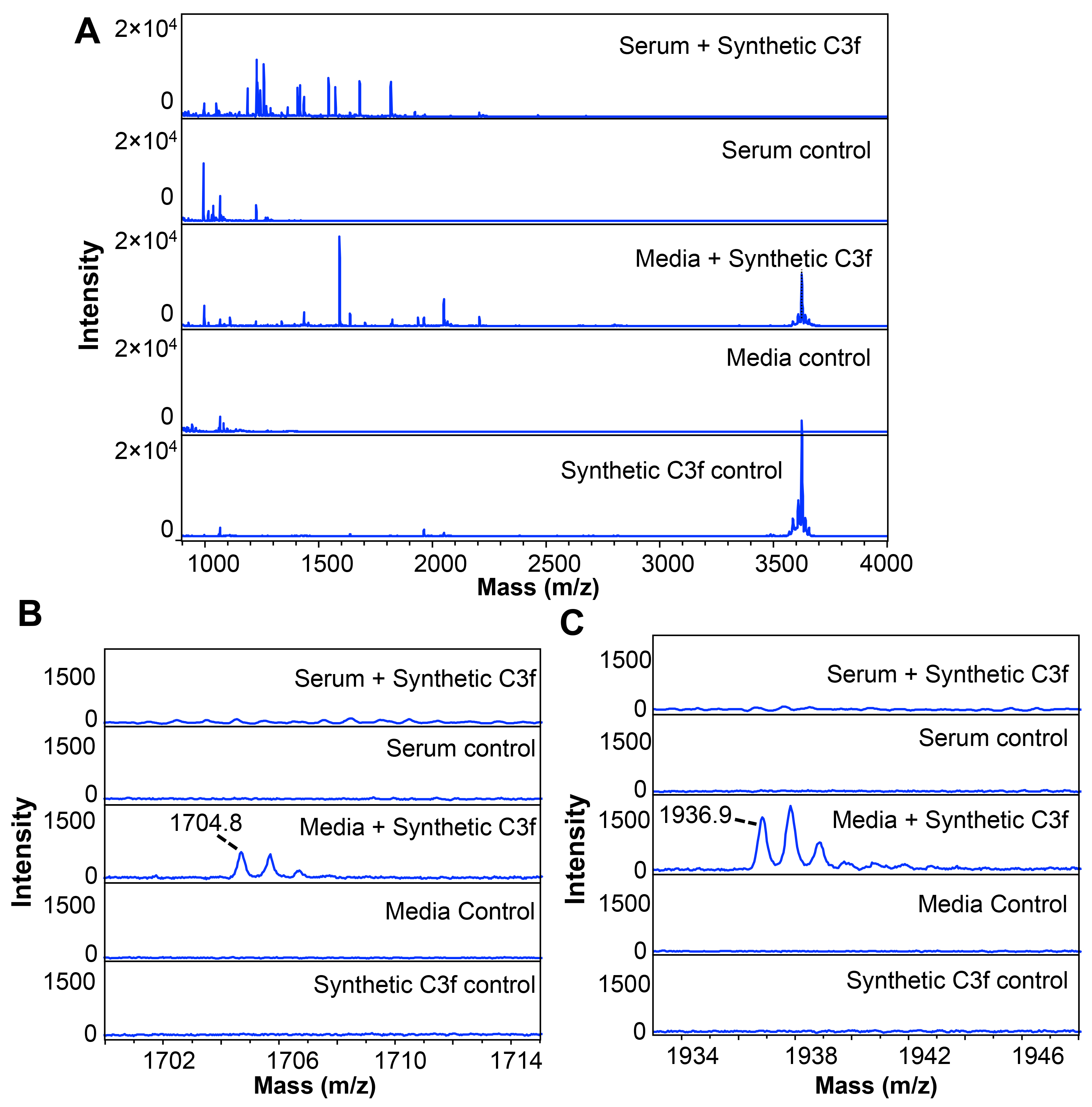


Figure S3 | MALDI-TOF MS spectra identifying proteolytic sites in synthetic C3f generated by mouse sera or HeyA8-MDR-conditioned media. (A) Wide-range view of the MALDI-TOF MS spectra showing C3f peaks. (B) MS spectra of the fragment with m/z=1704.8 (6xHis-SSATTFRL) and (C) the fragment with m/z=1936.9 (LWENGNLLR-6xHis), generated when synthetic C3f is incubated with HeyA8-MDR-conditioned media, but not with mouse serum.


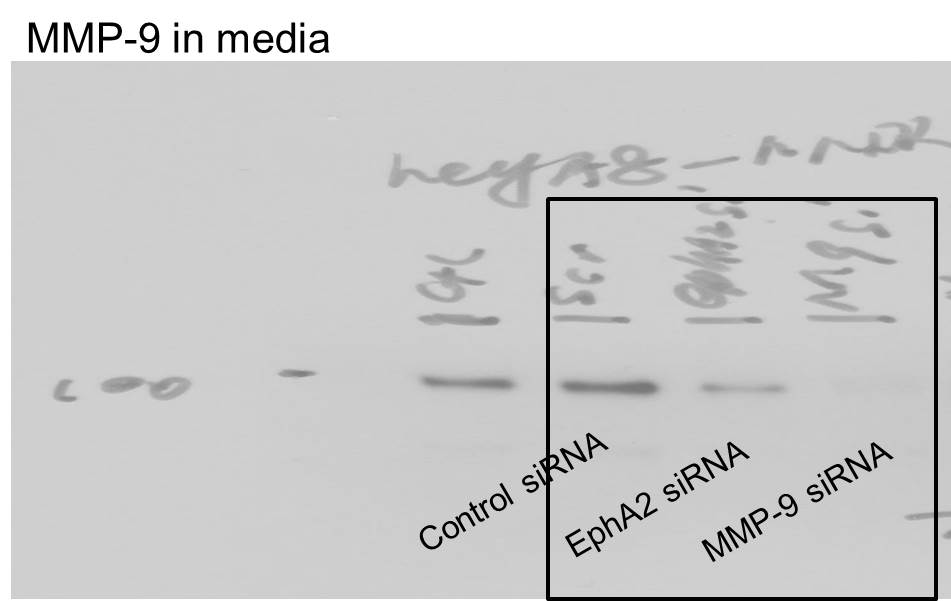


Figure S4 | Confirmation of the MMP9 expression level in conditioned culture media of cells which were respectively transfected with control siRNA, EphA2 siRNA or MMP9 siRNA using western blots-- Full blot image


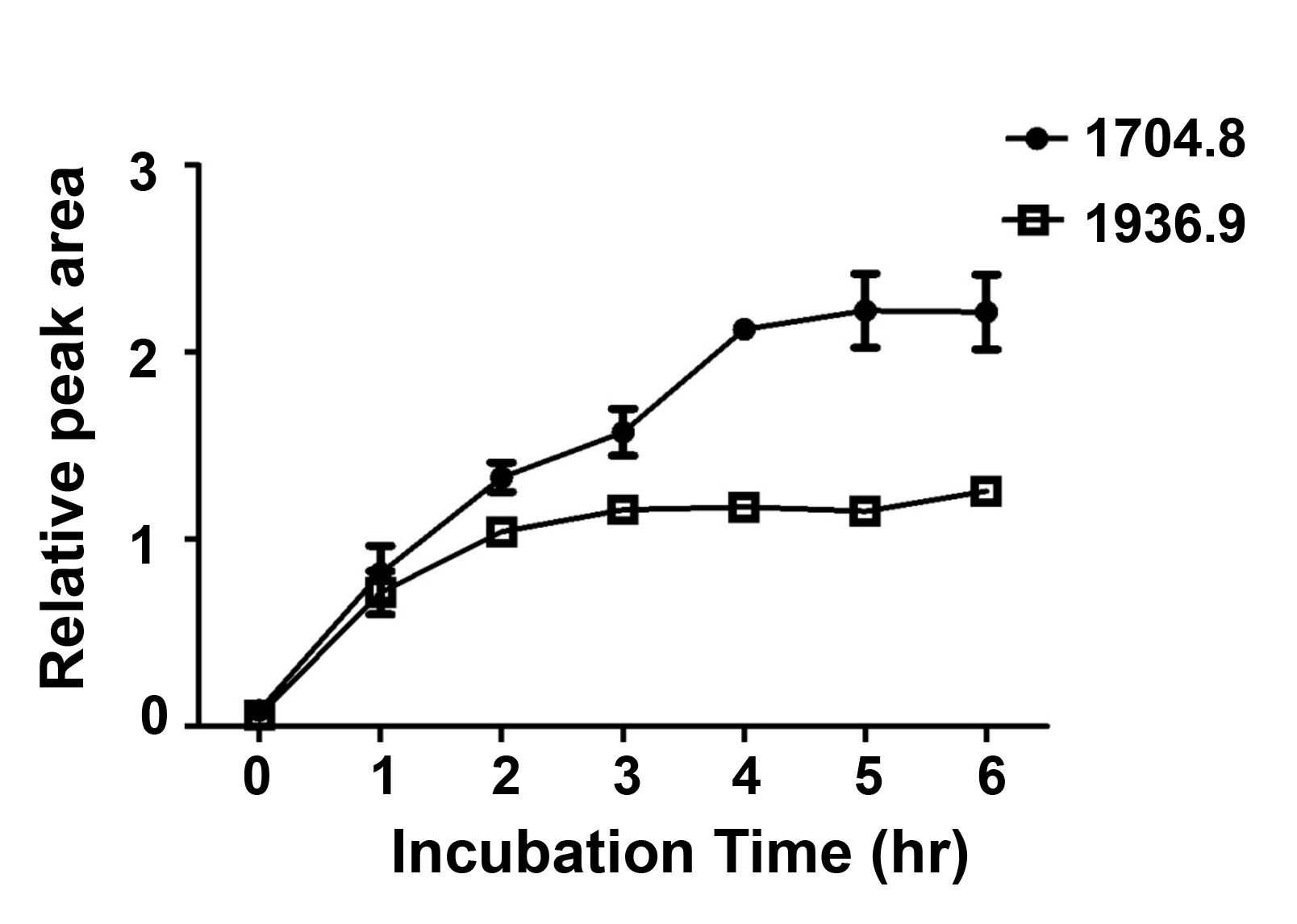


Figure S5 | 6xHis-SSATTFRL and LWENGNLLR-6xHis are specific proteolytic products generated when synthetic C3f was incubated with conditioned media. Relative peak areas of 6xHis-SSATTFRL and LWENGNLLR-6xHis generated after incubation of synthetic C3f with conditioned medium were plotted against time in hours. Complete cleavage of synthetic C3f was achieved within 4 hours.

Table S3 Intraday reproducibility of on-chip fractionation

|  | Sample1 | | | | Sample2 | | | | Sample3 | | | |
| --- | --- | --- | --- | --- | --- | --- | --- | --- | --- | --- | --- | --- |
| Fragment | N | Mean | SD | %CV | N | Mean | SD | %CV | N | Mean | SD | %CV |
| 1060.67 | 3 | 6681.65 | 636.15 | 9.52 | 3 | 6581.87 | 561.58 | 8.53 | 3 | 5301.80 | 249.12 | 4.70 |
| 1507.93 | 3 | 304.27 | 38.52 | 12.66 | 3 | 330.07 | 11.55 | 3.50 | 3 | 324.88 | 15.74 | 4.84 |
| 1608.98 | 3 | 355.64 | 20.95 | 5.89 | 3 | 359.50 | 25.31 | 7.04 | 3 | 405.92 | 42.11 | 10.37 |
| 2010.21 | 3 | 179.06 | 17.81 | 9.94 | 3 | 111.75 | 1.62 | 1.45 | 3 | 108.48 | 24.51 | 22.59 |
| 2081.25 | 3 | 489.62 | 10.96 | 2.24 | 3 | 350.93 | 18.98 | 5.41 | 3 | 389.60 | 21.40 | 5.49 |

Table S4 Interday reproducibility of on-chip fractionation

|  | Sample1 | | | | Sample2 | | | | Sample3 | | | |
| --- | --- | --- | --- | --- | --- | --- | --- | --- | --- | --- | --- | --- |
| Fragment | N | Mean | SD | %CV | N | Mean | SD | %CV | N | Mean | SD | %CV |
| 1060.67 | 9 | 6662.18 | 1072.69 | 16.10 | 9 | 6287.06 | 983.51 | 15.64 | 9 | 5819.29 | 1480.93 | 25.45 |
| 1507.93 | 9 | 273.17 | 64.36 | 23.56 | 9 | 250.83 | 69.25 | 27.61 | 9 | 241.54 | 54.80 | 22.69 |
| 1608.98 | 9 | 307.34 | 58.37 | 18.99 | 9 | 250.33 | 73.51 | 29.36 | 9 | 302.50 | 87.70 | 28.99 |
| 2010.21 | 9 | 143.95 | 35.55 | 24.69 | 9 | 83.09 | 22.90 | 27.56 | 9 | 121.08 | 27.56 | 22.76 |
| 2081.25 | 9 | 379.54 | 86.76 | 22.86 | 9 | 261.21 | 75.62 | 28.95 | 9 | 347.36 | 55.23 | 15.90 |


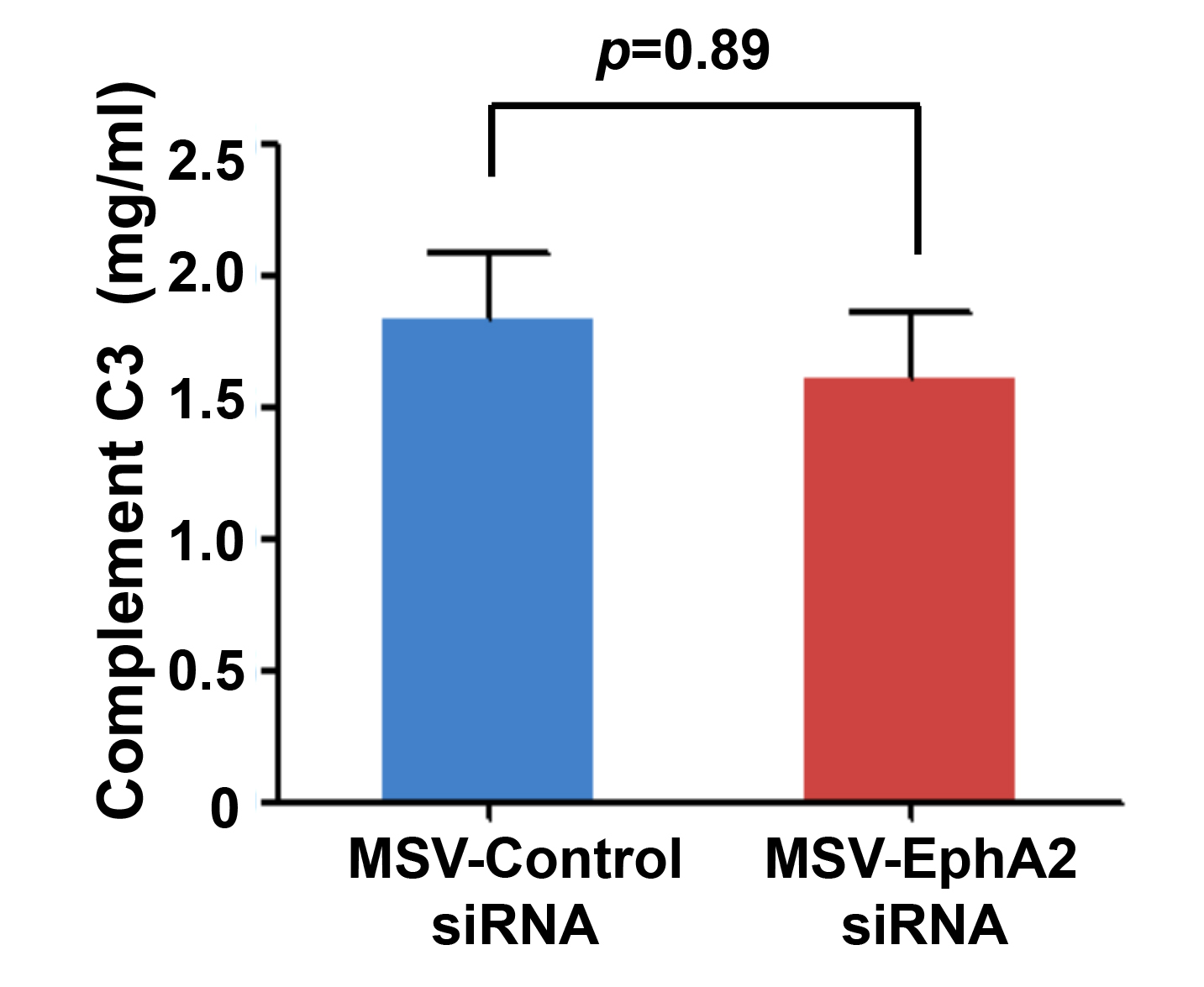


Figure S6 | Levels of complement C3 in sera of tumor-bearing mice, measured by ELISA, showed no statistically significant differences between MSV-Control siRNA and MSV-EphA2 siRNA treatments.
